# Utilizing prior-data-fitted networks and in-context learning: a transformer-based tabular foundation model for predicting symptomatic intracranial hemorrhage after successful recanalization

**DOI:** 10.3389/fneur.2025.1698741

**Published:** 2026-01-12

**Authors:** Zheng Li, Jiayue Zhang, Chengbing Wang, Hui Wang, Penglun Sun, Fenglin Chen, Xiaohua Shi, Zhongxin Xu

**Affiliations:** 1 Department of Neurology, China-Japan Union Hospital of Jilin University, Changchun, China; 2 Department of Vascular Surgery, Xuanwu Hospital of Capital Medical University, Beijing, China

**Keywords:** acute ischemic stroke, endovascular thrombectomy, predictive model, symptomatic intracranial hemorrhage, tabular prior-data fitted network

## Abstract

**Background and purpose:**

Symptomatic intracranial hemorrhage (sICH) is a serious complication after endovascular thrombectomy (EVT) and is strongly associated with poor outcomes in acute ischemic stroke. Existing risk scores show limited predictive accuracy. This study aims to develop and externally validate a transformer-based model for predicting sICH following successful recanalization in anterior circulation large vessel occlusion.

**Methods:**

A total of 661 EVT-treated patients were retrospectively analyzed as the derivation cohort, and 261 patients from another tertiary center were included as the external test cohort. A tabular prior-data-fitted network (TabPFN), a transformer-based foundation model, was constructed using angiographic biomarkers (basal ganglia blush, early venous filling), baseline ASPECT score, fasting blood glucose, collateral status, and the number of retriever passes. Logistic regression and XGBoost were also developed. Model performance was evaluated using the area under the receiver operating characteristic curve (AUC), precision, recall, and F1 score, and subsequently compared with established scores (ASIAN, TAG, IER-sICH).

**Results:**

In internal validation, TabPFN achieved an AUC of 0.948, comparable to XGBoost (0.953) and logistic regression (0.944), but superior to ASIAN (0.786), IER-sICH (0.687), and TAG (0.670). In external validation, TabPFN demonstrated the highest AUC (0.955), significantly outperforming existing scores (all *p* < 0.05), and exhibited the best F1 score and precision across cohorts.

**Conclusion:**

The TabPFN model effectively predicts the risk of sICH in Chinese stroke cohorts, enabling real-time risk stratification for antithrombotic therapy and postoperative blood pressure management.

## Introduction

Advances in endovascular thrombectomy (EVT) techniques have markedly improved clinical outcomes for patients with acute ischemic stroke (AIS) due to large vessel occlusion (LVO) (1, 2). However, despite successful post-EVT recanalization, a substantial proportion of patients continue to experience unfavorable prognoses attributable to postoperative symptomatic intracranial hemorrhage (sICH) (3, 4). Accordingly, accurate prediction of sICH risk is essential for improving postprocedural management, guiding therapeutic decisions, and customizing patient-specific monitoring protocols.

Current risk stratification tools, including the ASIAN score, TAG score, and IER-SICH nomogram (5–7), primarily employ logistic regression models based on baseline clinical and imaging variables, including the Alberta Stroke Program Early CT Score (ASPECTS), blood glucose levels, collateral circulation status, and onset-to-groin puncture time. Although these models provide valuable insights, their discriminative performance remains limited, with C-statistics ranging from 0.51 to 0.61, particularly in external validation cohorts (8). This limitation may be attributed to two primary challenges: (1) the assumption of linearity, which fails to adequately capture complex non-linear interactions (9), and (2) the exclusion of dynamic procedural variables, including the number of thrombectomy attempts and hemodynamic reperfusion status, which are critical determinants of hemorrhage risk (10, 11).

Recent advancements in deep learning (DL) methodologies offer promising solutions. A prior study successfully developed a three-dimensional convolutional neural network (3D-CNN) model based on post-thrombectomy dual-energy computed tomography (CT), which demonstrated excellent performance in distinguishing hemorrhagic transformation, achieving an area under the curve (AUC) of 0.911 (12). The implementation of such models remains controlled by the requirement of specialized imaging protocols and substantial computational resources, limiting their widespread adoption in clinical practice. Moreover, traditional machine learning (ML) algorithms typically require extensive datasets for effective training, which poses a significant challenge due to the low incidence rate of sICH (13). A tabular prior-data-fitted network (TabPFN), a transformer-based foundation model, offers a potential solution to these limitations through two key innovations: (1) prior-data fitting, which involves pre-training on synthetically generated tabular data to capture universal statistical patterns, and (2) in-context learning, which enables dynamic retrieval of analogous patterns from memory without requiring model retraining (14, 15). These advancements allow the model to deliver rapid and accurate predictions on small clinical datasets without the need for manual optimization.

The model’s performance was evaluated using multicenter data against existing risk scores (ASIAN, TAG, and IER-SICH nomogram) and ML models (XGBoost and logistic regression). This study built and validated a TabPFN-based model to predict sICH in patients achieving successful reperfusion [modified Thrombolysis in Cerebral Infarction (mTICI) 2b-], thereby enhancing post-EVT management and mitigating hemorrhagic complications in various clinical settings.

## Methods

This study was approved by the Institutional Review Boards of the China-Japan Union Hospital of Jilin University (No. 2025042416) and Xuanwu Hospital of Capital Medical University (No. 2022054). The requirement for informed consent was waived due to the retrospective nature of the study.

### Patient selection

The derivation cohort comprised consecutive patients with anterior circulation LVO-AIS who underwent EVT at a single stroke center from January 2017 to December 2024. The external validation cohort included consecutive patients with anterior circulation LVO-AIS who underwent EVT in another tertiary stroke center between January 2022 and December 2024. Inclusion criteria were: (1) age ≥18 years; (2) without evidence of hemorrhage on preoperative brain CT; (3) occlusion of the internal carotid artery (ICA) or the M1 segment of middle cerebral artery (MCA) confirmed by computed tomographic angiography (CTA), magnetic resonance angiography (MRA), or digital subtraction angiography (DSA); and (4) EVT performed within 24 h of symptom onset. Exclusion criteria were: (1) patients treated with devices other than stent-retriever or contact aspiration; (2) modified treatment in cerebral infarction scale (mTICI) scores of 0–2a after EVT; and (3) patients with missing clinical or imaging data.

### Data collection

Baseline clinical data were collected, including age, sex, comorbidities (hypertension, diabetes mellitus, and atrial fibrillation), laboratory tests [fasting blood glucose (FGB), low-density lipoprotein (LDL), creatinine (Cr), and blood urea nitrogen (BUN)], intravenous thrombolysis (IVT), admission National Institutes of Health Stroke Scale score (NIHSS), Acute Stroke Prognosis Early CT (ASPECT) scores, stroke subtype classified according to the Trial of Org 10,172 in Acute Stroke Treatment (TOAST) classification system, time from stroke onset to puncture (OTP), and time from stroke onset to recanalization (OTR). EVT treatment details, including occlusion site, number of retriever passes, first-line thrombectomy device (contact aspiration or stent retriever), collateral circulation, basal ganglia blush (BGB) sign, and early venous filling (EVF) sign, were also collected for analysis. Collateral status was evaluated using the American Society of Interventional and Therapeutic Neuroradiology (ASITN)/Society of Interventional Radiology (SIR) Collateral Flow Grading System, with good collateral status defined as an ASITN/SIR score of ≥2 (16). The BGB is defined as an increased contrast enhancement of the basal ganglia in post-recanalization angiograms (11). The EVF is defined as the appearance of contrast agent opacification in cortical veins and lenticulostriate veins prior to the venous phase (10). Two independent reviewers evaluated the BGB, EVF, and ASITN/SIR scores, with blinding to the occurrence of sICH. Discrepancies were resolved through consensus between the two reviewers. For statistical analysis, etiology was categorized into three groups: large-artery atherosclerosis, cardioembolic, and others (including small-vessel occlusion, other determined etiologies, and undetermined etiologies).

### Definition of sICH

Follow-up non-contrast CT or MRI was performed 48 h after the procedure, or immediately in the event of clinical deterioration. Hemorrhagic transformation was classified according to the European Cooperative Acute Stroke Study III (ECASS III) criteria as either hemorrhagic infarction (HI1, HI2) or parenchymal hematoma (PH1, PH2) (17). sICH was defined as imaging evidence of hemorrhagic transformation accompanied by an increase in NIHSS of ≥4 points, coma (Glasgow coma scale ≤ 8), and/or death, which could be attributed solely to stroke edema (17).

### Model fitting and evaluation

**Figure 1:**
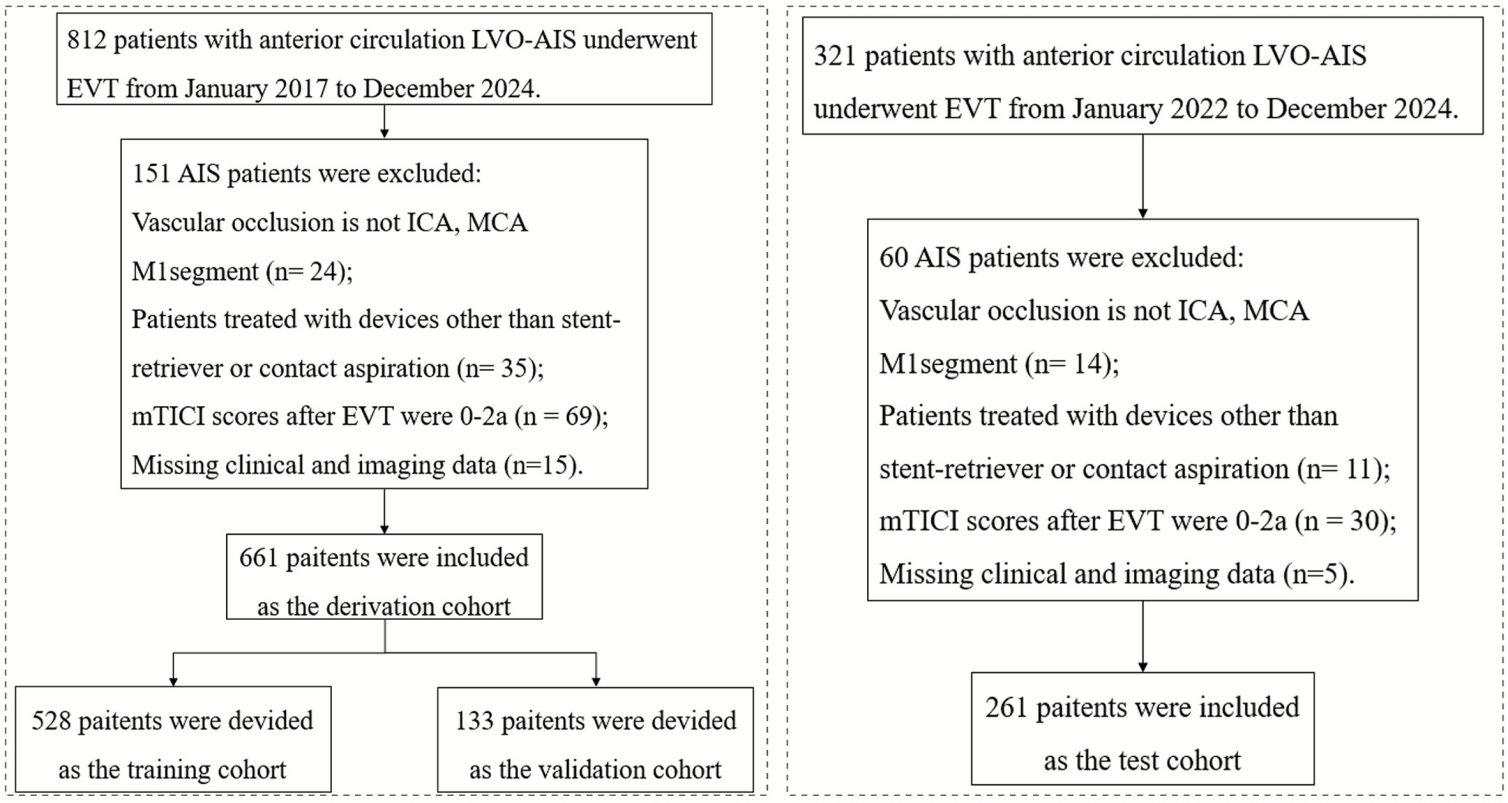
Flowchart of patient selection and cohort assignment.

**Figure 2:**
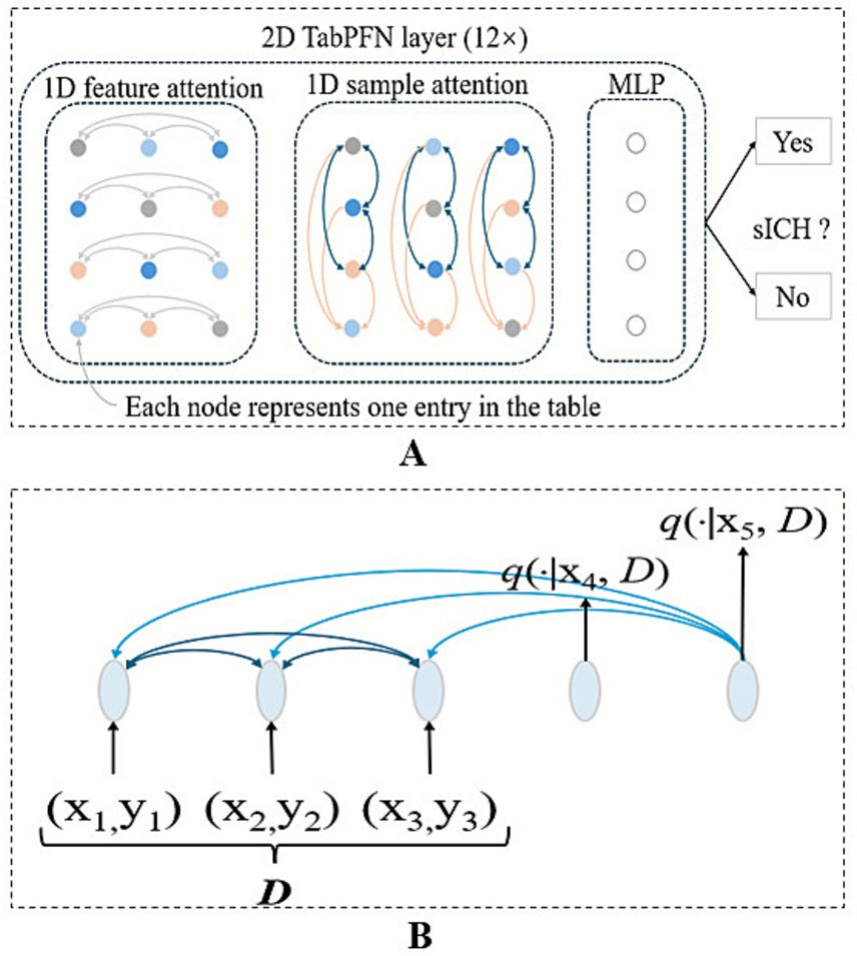
Architecture of the TabPFN predictive model. **(A)** TabPFN employs a bidirectional attention mechanism: Intra-row attention captures the correlations among various features within a single sample, while inter-column attention learns the patterns of the same feature across multiple samples. **(B)** The model also incorporates attention mechanisms between samples in the context and between the context and the query, enabling more effective representation learning.

The least absolute shrinkage and selection operator (LASSO) algorithm, implemented through five-fold cross-validation, was employed to identify variables demonstrating the strongest predictive power for the target outcome. This approach enhanced the model’s interpretability by eliminating redundant features and improved its practical applicability in clinical settings. The TabPFN, a transformer-based foundation model pretrained on ~100 million synthetic datasets generated through structural causal models, which was called “prior-data fitting” (Figure 1) (14). The TabPFN model primarily utilizes “In-Context Learning” as its inference mechanism. In this approach, the “training” data, denoted as (x_train, y_train), is provided to the model as contextual input. The model then conducts a single forward-pass inference using this context together with the new data (x_validation,?) to generate predictions for the corresponding classes (y_validation). Importantly, the parameters of the TabPFN model remain unaltered. Methodologically, in-context learning was implemented using TabPFNClassifier.fit() function for,^1^ without any fine-tuning. This approach processed the training data as contextual input during a single forward pass without parameter updates, enabling rapid prediction. The XGBoost library (version 2.1.2) for the XGBoost model and Scikit-learn (version 1.5.2) for the logistic regression model were used. The derivation cohort was randomly divided into training and validation sets in an 8:2 ratio using stratified sampling to preserve the proportion of sICH cases. The external test cohort was used to externally validate the robustness and generalization of the TabPFN model. In addition, XGBoost and logistic regression models were developed under two conditions: with class imbalance correction using the synthetic minority oversampling technique (SMOTE) algorithm (positive: 0.5 vs. negative: 1), and without class imbalance correction. For the TabPFN model, the “balance_probabilities” parameter was set as True to address the class imbalance (see Figure 2).

### Statistical analysis

All statistical analyses and model construction were performed using R software (Version 4.3.1) and Python (Version 3.9.1). Baseline clinical characteristics were examined and compared between the derivation and test cohorts. Results were reported as frequency (percentage) for categorical variables and as mean ± standard deviation (SD) or median (interquartile range, IQR) for continuous variables, depending on the normality of distribution. The categorical variables were compared using *χ*^2^ tests or Fisher’s exact tests. Continuous variables were compared using the Student’s *t*-test (for normally distributed data) or the Mann–Whitney *U* test (for non-normally distributed data). The value of *p* < 0.05 was considered statistically significant.

The accuracy, AUC, F1 score, negative predictive value (NPV), positive predictive value (PPV), precision, recall, and specificity of the TabPFN model for predicting sICH were calculated. Calibration curves assessed the agreement between predicted probabilities and actual outcomes. Decision curve analysis (DCA) quantified the model’s clinical benefit, while SHAP analysis was used to interpret feature contributions at both populations. The TabPFN model was compared with the previously published risk models, including the ASIAN score, TAG score, and the sICH nomogram. The ASIAN score assigns: 2 points for onset-to-groin puncture time >270 min, 3 points for passes with retriever >3, 4 points for poor collateral circulation, 1 point for ASPECTS 6 to 7, 3 points for ASPECTS <6, 1 point for baseline glucose level 7.1–11.1 mmol/L, and 4 points for baseline glucose level ˃11.1 mmol/L. The TAG score assigns: 2 points for thrombolysis in cerebral ischemia 0 to 2a, 2 points for ASPECTS 6 to 7, 4 points for ASPECTS <6, and 1 point for baseline glucose level ≥8.3 mmol/L. The sICH nomogram is a continuous scoring system incorporating the NIHSS score, onset-to-end procedure time, age, recanalization status, and collateral circulation.

## Results

### Patient characteristics

The baseline characteristics of the derivation (*N* = 661) and test (*N* = 261) cohorts are summarized in Table 1. The median age was significantly higher in the derivation cohort (66 years, IQR: 59–74) compared with the test cohort (65 years, IQR: 58–71; *p* = 0.027). The sICH was observed in 9.1% (*n* = 61) and 7.7% (*n* = 20) of the patients in the derivation and external test cohorts (*p* = 0.492), respectively. Significant differences were detected in median baseline ASPECT score (*p* < 0.001), EVT device type (*p* = 0.012), creatinine levels (*p* = 0.007), and onset-to-reperfusion time (*p* < 0.001). No significant differences were noted for other baseline variables.

**Table 1:** Patient demographics and baseline characteristics.

Characteristic	Derivation Cohort, *n* = 661^a^	Test cohort, *n* = 261^a^	*p*-value
Age	66 (59, 74)	65 (58, 71)	0.027^b^
Sex (male)	397 (60.1%)	151 (57.9%)	0.539^c^
Hypertension	329 (49.8%)	119 (45.6%)	0.253^c^
Diabetes	153 (23.1%)	70 (26.8%)	0.241^c^
Atrial fibrillation	307 (46.4%)	104 (39.8%)	0.069^c^
Baseline NIHSS score	16.0 (14.0, 18.0)	16.0 (14.0, 18.0)	0.083^b^
Baseline ASPECT score	9.00 (8.00, 10.00)	8.00 (7.00, 9.00)	<0.001^b^
TOAST classification			0.151^c^
Others	89 (13.5%)	28 (10.7%)	
Cardioembolic	363 (54.9%)	134 (51.3%)	
Atherosclerotic	209 (31.6%)	99 (37.9%)	
IVT	157 (23.8%)	76 (29.1%)	0.091^c^
FBG	7.45 (5.79, 9.05)	6.90 (5.80, 8.70)	0.177^b^
LDL	2.44 (1.97, 2.97)	2.50 (2.20, 2.70)	0.869^b^
BUN	6.07 (4.64, 7.31)	6.20 (5.30, 7.10)	0.072^b^
Cr	90 (78, 101)	88 (78, 94)	0.007^b^
sICH	60 (9.1%)	20 (7.7%)	0.492^c^
Hemorrhagic transformation			0.712^c^
HI 1	90.0 (13.6%)	37.0 (14.2%)	
HI 2	74.0 (11.2%)	25.0 (9.6%)	
PH 1	55.0 (8.3%)	15.0 (5.7%)	
PH 2	48.0 (7.3%)	17.0 (6.5%)	
BGB	140 (21.2%)	48 (18.4%)	0.344^c^
EVF	91 (13.8%)	43 (16.5%)	0.293^c^
OTP	277 (246, 313)	276 (244, 320)	0.811^b^
OTR	362 (318, 400)	343 (317, 383)	<0.001^b^
Collateral status			0.638^c^
Good	333 (50.4%)	127 (48.7%)	
Poor	328 (49.6%)	134 (51.3%)	
The first-line thrombectomy device			0.012^c^
SR	455 (68.8%)	157 (60.2%)	
CA	206 (31.2%)	104 (39.8%)	
Occlusion site			0.250^c^
MCA	365 (55.2%)	155 (59.4%)	
ICA	296 (44.8%)	106 (40.6%)	
Passes of retriever_ > 3	82 (12.4%)	27 (10.3%)	0.383^c^

a Median (IQR); *n* (%).

b Wilcoxon rank-sum test.

c Pearson’s chi-squared test.

NIHSS, National Institutes of Health Stroke Scale score; ASPECTS, Acute stroke prognosis early CT Score; OTP, Onset to groin puncture time; OTR, Onset to recanalization; ICA, Internal carotid artery; MCA, Middle cerebral artery; FBG, Fasting blood glucose; BUN, Blood urea nitrogen; Cr, Creatinine; LDL, Low-density lipoprotein; IVT, Intravenous thrombolysis; sICH, Symptomatic intracerebral hemorrhage; CA, Contact aspiration; SR, Stent retriever; HI, Hemorrhagic infarction; PH, Parenchymal hematoma.

### Model performance in the internal validation cohort

**Figure 3:**
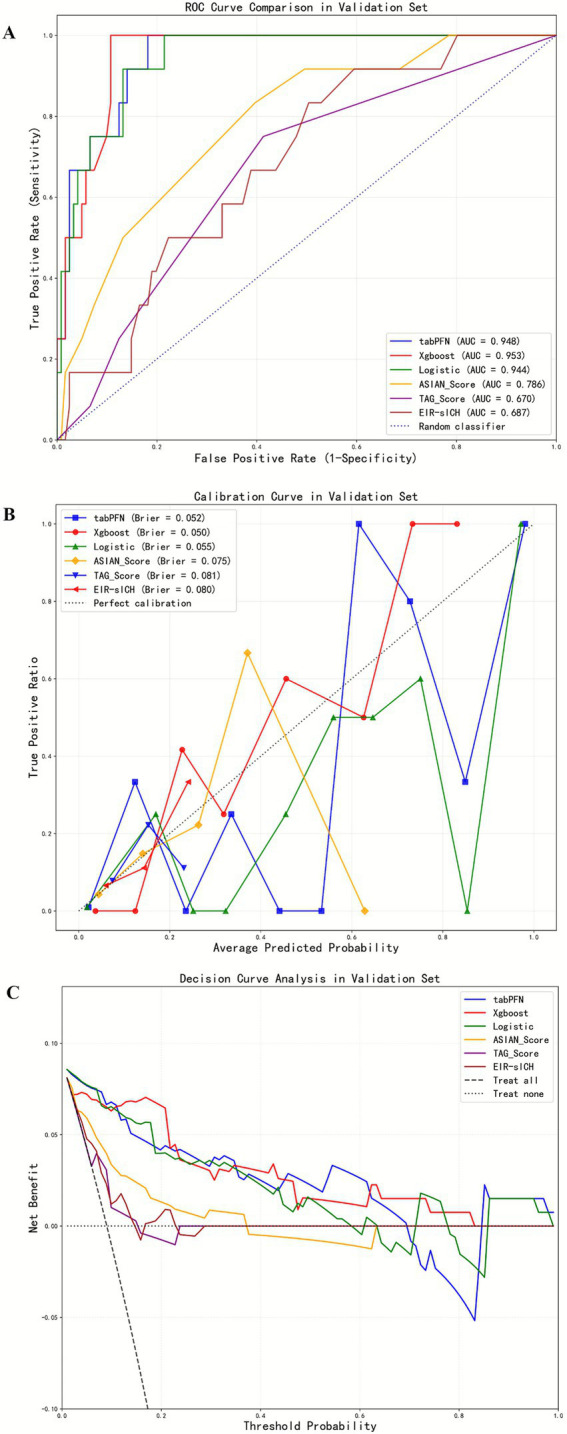
Comparison of the six models without class-imbalance correction in the ROC curves, calibration curves, and decision curve analysis across the validation cohort **(A–C)**.

After feature selection by LASSO, basal ganglia blush, early venous filling, baseline ASPECT score, collateral status, and retriever pass count were retained. Without class imbalance correction in the internal validation cohort, the TabPFN model demonstrated an AUC of 0.948 for predicting sICH, accuracy of 0.932, F1 score of 0.64, NPV of 0.967, PPV of 0.615, precision of 0.615, recall of 0.667, and specificity of 0.959 (Table 2 and Figure 3). In comparison, the XGBoost model exhibited an AUC of 0.953, with an accuracy of 0.752, F1 score of 0.421, NPV of 1, PPV of 0.267, precision of 0.267, recall of 1, and specificity of 0.727. The logistic regression model exhibited an AUC of 0.944, with an accuracy of 0.857, F1 score of 0.486, NPV of 0.972, PPV of 0.36, precision of 0.36, recall of 0.75, and specificity of 0.868 (Table 2).

**Table 2:** Performance metrics of predictive models without class-imbalance correction.

Model	Accuracy	AUC	Precision	Specificity	Recall	F1-Score	PPV	NPV
The internal validation cohort								
tabPFN	0.932	0.948	0.615	0.959	0.667	0.640	0.615	0.967
XGBoost	0.752	0.953	0.267	0.727	1.000	0.421	0.267	1.000
Logistic	0.857	0.944	0.360	0.868	0.750	0.487	0.360	0.972
ASIAN_Score	0.624	0.786	0.172	0.603	0.833	0.286	0.172	0.973
TAG_Score	0.857	0.670	0.111	0.934	0.083	0.095	0.111	0.911
EIR-sICH	0.526	0.687	0.141	0.496	0.833	0.241	0.141	0.968
The external test cohort								
tabPFN	0.935	0.955	0.556	0.950	0.750	0.638	0.556	0.979
XGBoost	0.720	0.931	0.202	0.705	0.900	0.330	0.202	0.988
Logistic	0.877	0.949	0.364	0.884	0.800	0.500	0.364	0.982
ASIAN_Score	0.636	0.763	0.143	0.627	0.750	0.240	0.143	0.968
TAG_Score	0.831	0.688	0.147	0.880	0.250	0.185	0.147	0.934
EIR-sICH	0.536	0.660	0.115	0.519	0.750	0.199	0.115	0.962

After class imbalance correction in the internal validation cohort, the TabPFN model demonstrated an AUC of 0.948 for predicting sICH, with an accuracy of 0.834, F1 score of 0.5, NPV of 0.917, PPV of 0.34, precision of 0.34, recall of 0.92, and specificity of 0.826. The XGBoost model exhibited an AUC of 0.944, with an accuracy of 0.81, F1 score of 0.511, NPV of 0.957, PPV of 0.352, precision of 0.352, recall of 0.932, and specificity of 0.807. The logistic regression model exhibited an AUC of 0.945, with an accuracy of 0.864, F1 score of 0.5, NPV of 0.972, PPV of 0.375, precision of 0.375, recall of 0.75, and specificity of 0.876.

The ASIAN score exhibited an AUC of 0.786, with an accuracy of 0.624, F1 score of 0.286, NPV of 0.973, PPV of 0.172, precision of 0.172, recall of 0.833, and specificity of 0.603. The sICH nomogram achieved an AUC of 0.687, with an accuracy of 0.526, F1 score of 0.241, NPV of 0.968, PPV of 0.141, precision of 0.141, recall of 0.833, and specificity of 0.496. Lastly, the TAG score recorded an AUC of 0.67, with an accuracy of 0.857, F1 score of 0.095, NPV of 0.911, PPV of 0.111, precision of 0.111, recall of 0.083, and specificity of 0.934 (Table 2).

### Model performance in the external test cohort

**Figure 4:**
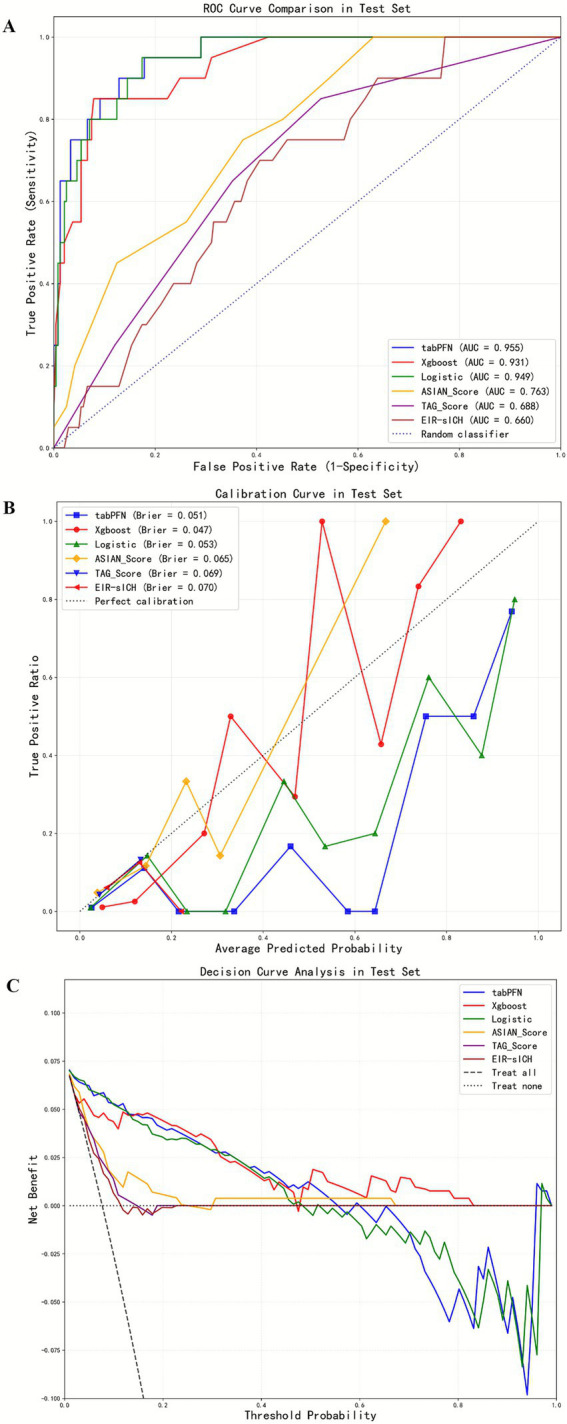
Comparison of the six models without class-imbalance correction in the ROC curves, calibration curves, and decision curve analysis across the test cohort **(A–C)**.

Without class imbalance correction in the external test cohort, the TabPFN model demonstrated a robust predictive capability for sICH, evidenced by an AUC of 0.955, accuracy of 0.935, F1 score of 0.638, NPV of 0.979, PPV of 0.556, precision of 0.556, recall of 0.75, and specificity of 0.95 (Table 2 and Figure 4). In comparison, the XGBoost model exhibited an AUC of 0.931, with an accuracy of 0.72, F1 score of 0.33, NPV of 0.988, PPV of 0.202, precision of 0.202, recall of 0.9, and specificity of 0.705. The logistic regression model exhibited an AUC of 0.949, with an accuracy of 0.877, F1 score of 0.5, NPV of 0.982, PPV of 0.364, precision of 0.364, recall of 0.8, and specificity of 0.884 (Table 2).

After class imbalance correction in the external test cohort, the TabPFN model demonstrated an AUC of 0.954 for predicting sICH, with an accuracy of 0.839, F1 score of 0.46, NPV of 0.967, PPV of 0.31, precision of 0.31, recall of 0.9, and specificity of 0.834. The XGBoost model exhibited an AUC of 0.937, with an accuracy of 0.781, F1 score of 0.447, NPV of 0.95, PPV of 0.297, precision of 0.297, recall of 0.91, and specificity of 0.79. The logistic regression model exhibited an AUC of 0.949, with an accuracy of 0.892, F1 score of 0.548, NPV of 0.986, PPV of 0.404, precision of 0.404, recall of 0.85, and specificity of 0.896.

The ASIAN score reached an AUC of 0.763, with an accuracy of 0.636, F1 score of 0.24, NPV of 0.968, PPV of 0.143, precision of 0.143, recall of 0.75, and specificity of 0.627. The sICH nomogram yielded an AUC of 0.66, alongside an accuracy of 0.536, F1 score of 0.199, NPV of 0.962, PPV of 0.114, precision of 0.114, recall of 0.75, and specificity of 0.519. Meanwhile, the TAG score recorded an AUC of 0.688, with an accuracy of 0.831, F1 score of 0.185, NPV of 0.934, PPV of 0.147, precision of 0.147, recall of 0.25, and specificity of 0.88 (Table 2).

### Comparison with the previously published risk models

The calibration curves were shown in Figures 4B,C. In the internal validation cohort, the Brier scores for TabPFN, XGBoost, logistic regression, ASIAN score, TAG score, and EIR-sICH nomogram were 0.052, 0.05, 0.055, 0.075, 0.081, and 0.08, respectively. In the external test cohort, the Brier scores for TabPFN, XGBoost, logistic regression, ASIAN score, TAG score, and EIR-sICH nomogram were 0.051, 0.047, 0.053, 0.065, 0.069, and 0.07, respectively. The decision curves revealed that the TabPFN model consistently provided greater net benefits across a wide range of threshold probabilities, suggesting its clinical usefulness (Figures 3,4).

Delong’s test was conducted to compare the AUC, demonstrating that the TabPFN model exhibited comparable performance to the logistic regression model (0.955 vs. 0.949, *p* = 0.055), while significantly outperforming the XGBoost model (0.955 vs. 0.931, *p* = 0.019), ASIAN score (0.955 vs. 0.763, *p* < 0.001), sICH nomogram (0.955 vs. 0.66, *p* < 0.001), and TAG score (0.955 vs. 0.688, *p* < 0.001) in the external test cohort (Table 2 and Figure 4). In the internal validation cohort, the TabPFN model showed comparable performance to the logistic regression model (0.948 vs. 0.944, *p* = 0.472) and the XGBoost model (0.948 vs. 0.953, *p* = 0.716), while outperforming the ASIAN score (0.948 vs. 0.786, *p* = 0.008), the sICH nomogram (0.948 vs. 0.687, *p* = 0.001), and the TAG score (0.948 vs. 0.67, *p* < 0.001) (Table 2 and Figure 3).

**Figure 5:**
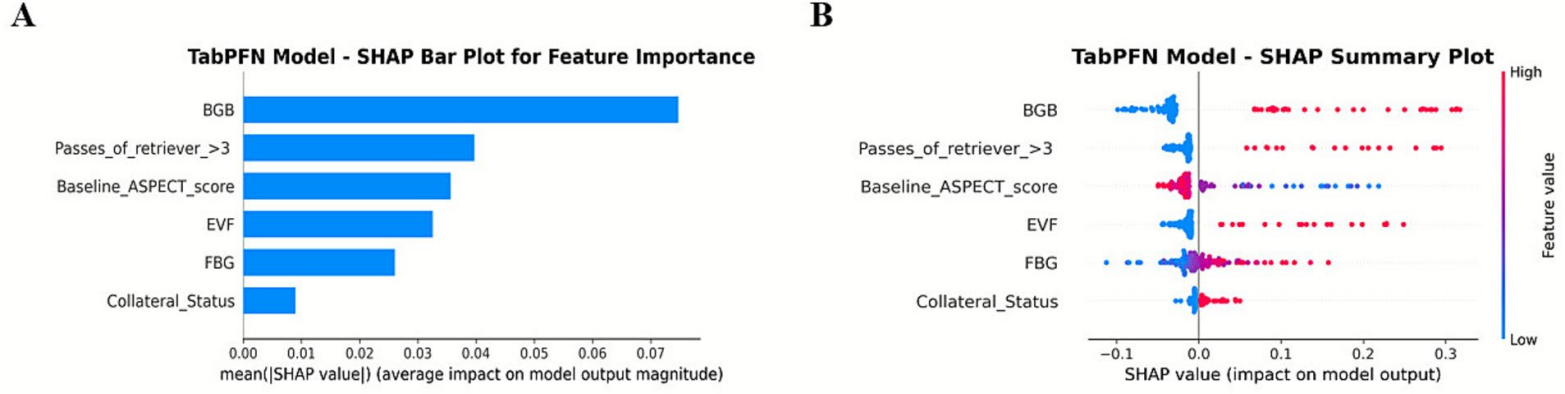
SHAP summary plot for global feature importance in the TabPFN model.

In Figure 5, BGB, the number of thrombectomy attempts, and the ASPECTS score are the three most significant features. The presence of BGB, a pass of retriever ˃3, a lower ASPECTS score, the presence of EVF, and poor collateral circulation were identified as significantly associated with an increased model-predicted probability of sICH occurrence.

## Discussion

The development of a robust predictive model for sICH following EVT is essential for optimizing post-procedural management, guiding therapeutic decisions, and customizing patient-specific monitoring protocols (18). This study employed the TabPFN model that exhibited superior discriminative performance (AUC: 0.948 in the validation cohort, 0.955 in the test cohort) compared with established risk scores such as ASIAN, TAG, and IER-SICH nomogram across both the validation and test cohorts. Delong’s test showed that the TabPFN model outperformed the XGBoost model (0.955 vs. 0.931, *p* = 0.019) in the external test cohort. Moreover, in the context of class imbalance, the F1 score and precision of the TabPFN model are all higher than those of the conventional ML models (XGBoost and logistic regression).

Both prior studies and the findings of this study indicated that established sICH prediction tools, such as the ASIAN score, TAG score, and IER-SICH nomogram, display limited generalizability in external validations (8). This limitation is attributed to several key factors: (1) Existing risk scales predominantly rely on static clinical parameters such as baseline glucose levels, ASPECTS, and procedural time, while neglecting dynamic angiographic biomarkers like BGB and EVF. These dynamic features act as direct indicators of blood–brain barrier disruption and hyperperfusion injury, which are critical mechanisms underlying the pathogenesis of sICH (10, 11). (2) Inability to effectively capture non-linear interactions. The linear weighting systems used by the ASIAN and TAG scores, along with the logistic regression framework of the IER-SICH nomogram, inherently constrain their capacity to model complex non-linear interactions. Clinically significant synergies, such as the exacerbated endothelial injury caused by hyperglycemia combined with multiple thrombectomy attempts, are systematically overlooked. (3) Misalignment between statistical significance and predictive utility (19, 20). Although variables in these tools are selected based on univariate or multivariate logistic regression, which emphasizes independent correlations with sICH, statistical significance does not necessarily equate to predictive value. Variables with marginal effect sizes (e.g., OR ≈1.1–1.3) may unnecessarily increase model complexity without enhancing discrimination, as evidenced by the modest AUCs (0.660–0.763) of traditional tools compared with the superior performance of TabPFN (AUC: 0.955).

In this study, TabPFN demonstrated superior discriminative performance compared with established risk scores. Importantly, even when restricted to the identical input variables used in the TabPFN model, the F1 score and precision of the TabPFN model remained higher than those of the logistic regression model (with or without class imbalance correction), confirming that its advantage stems from modeling non-linear interactions (e.g., hyperglycemia × multiple passes). Despite its potential, the tabPFN model exhibited modest performance in identifying the minority class (positive cases) under conditions of class imbalance (21). Internal and external validation yielded F1-scores approximating 0.65, with precision ranging from 0.55 to 0.65 and recall between 0.66 and 0.75. This performance pattern, characterized by moderate recall but lower precision and suboptimal F1-score, suggests that the model’s ability to effectively handle the inherent class imbalance remains limited. Interestingly, after applying the class imbalance correction, the precision, F1 score, and recall of the XGBoost and logistic regression models improved, whereas the precision and F1 score of the TabPFN model decreased. Therefore, a larger sample size is required to enhance the model’s capacity to learn and more accurately predict the positive category, specifically the occurrence of sICH.

The TabPFN model holds substantial promise for guiding post-EVT clinical decision-making by enabling accurate risk stratification for sICH. By identifying a high-risk phenotype characterized by biomarkers such as BGB and EVF, the model establishes a foundation for personalized management. Clinically, this study hypothesized that the risk stratification could inform specific management strategies, distinct from the “one-size-fits-all” protocols derived from conventional models like the IER-SICH nomogram. High-risk patients may benefit from stricter systolic blood pressure control (e.g., <140 mmHg) to mitigate reperfusion injury, a mechanism proposed by the strong predictive value of EVF in our model. For the high-risk subgroup, a more conservative approach to antithrombotic therapy (e.g., deferring aspirin or clopidogrel for 24–48 h) could be considered to minimize hemorrhagic conversion. In addition, prioritizing advanced neuroimaging, such as serial CT perfusion or MRI susceptibility-weighted imaging, could aid in the early detection of hemorrhagic transformation in this subgroup. It is important to note that the model provides precise risk probability, whereas the clinical effectiveness of these specific interventions must be confirmed in future randomized controlled trials.

Despite its potential, the clinical utility of the TabPFN model requires further validation. Prospective trials should evaluate TabPFN’s impact on delaying antithrombotic therapy in high-risk patients, confirming whether this strategy improves clinical outcomes without compromising the benefits of early recanalization. Moreover, it is imperative to consider the potential for TabPFN to overestimate risk in low-probability cases. In such patients, adopting a more conservative approach—such as postponing anticoagulant therapy—based on the high-risk predictions generated by the TabPFN model may adversely affect outcomes. Consequently, while the model can be a valuable tool in clinical practice, it should not be solely relied upon. Clinicians should integrate their professional judgment and experience with the predictive outputs of the TabPFN model to formulate appropriate treatment strategies.

The TabPFN framework introduces two major advancements in the prediction of sICH. First, its ability to process tabular data with minimal preprocessing effectively addresses a persistent challenge in applying ML to stroke care, which is often constrained by small sample sizes and missing data. Unlike traditional models such as logistic regression, which require complete datasets and manual feature engineering, the transformer-based architecture of TabPFN autonomously identifies predictive patterns even within sparse or heterogeneous inputs. Second, the integration of angiographic biomarkers (BGB and EVF) provides mechanistic insights into the pathophysiology of sICH (Figure 5). BGB, indicative of blood–brain barrier compromise, and EVF, suggestive of hyperperfusion injury, serve as direct markers of vascular fragility associated with hemorrhagic transformation (11). These features, often neglected in existing scoring systems, are consistent with recent evidence highlighting the significance of endothelial injury and reperfusion dynamics in the pathogenesis of sICH. Nevertheless, it is important to acknowledge the computational demands of TabPFN. While the model validates the efficiency in CPU-based inference, its pretraining phase requires substantial resources. Consequently, future research should incorporate cost-effectiveness and implementation analyses to evaluate the feasibility of real-world deployment.

In this study, the consistently high AUC (>0.94) observed in all three models, including standard logistic regression, highlights their strong discriminative performance. This finding is primarily attributable to the inclusion of angiographic biomarkers–specifically BGB and EVF–which serve as robust intra-procedural indicators of blood–brain barrier disruption and contrast extravasation. Their well-established association with sICH renders the prediction task more linear, thereby enabling even conventional logistic regression to perform well. Importantly, these biomarkers are clinically meaningful and available immediately after recanalization, allowing for timely clinical decision-making before the patient leaves the angiography suite. However, AUC alone provides an incomplete assessment of model utility, particularly in settings involving rare events. In our cohort, where sICH incidence is low (<10%), the TabPFN model achieved a superior F1 score compared to both Logistic Regression and XGBoost. This indicates that while strong predictive features can drive high AUC values in linear models, the TabPFN architecture is more effective at handling class imbalance by achieving a more favorable balance between precision and recall. Such performance is critical in clinical practice, where minimizing false negatives is essential to ensure early identification and intervention for high-risk patients.

### Limitations

Several limitations are noted in this study. (1) While the model demonstrated high discrimination (AUC), the calibration curve in the internal and external validation cohorts exhibited instability. This is likely due to the relatively small sample size of positive sICH cases (the occurrence of sICH <10%), which limits the valuation of calibration accuracy at specific probability thresholds. Clinicians should interpret the precise numerical probability with caution. (2) The retrospective design introduces potential selection bias, particularly due to the exclusion of patients with incomplete angiographic and clinical data. This limitation also undermines one of the major strengths of TabPFN, which is its capability to manage missing data effectively. (2) External validation was limited to a single Chinese center, thereby limiting the applicability to other populations. Future studies need to prioritize multinational cohorts (e.g., posterior circulation strokes, non-Asian populations). (3) Variables like post-EVT blood pressure variability and antithrombotic regimens were not included, which may refine risk stratification. Future prospective multicenter studies should address these gaps while exploring the integration of advanced imaging biomarkers and serum markers of endothelial dysfunction. (4) As this study excluded patients who experienced failed recanalization, the applicability of its TabPFN model to such cases remains uncertain.

## Conclusion

The TabPFN model, which integrates key angiographic biomarkers—BGB and EVF—with baseline ASPECT score, FBG, collateral circulation status, and number of retrieval passes, effectively predicts the risk of sICH in Chinese stroke cohorts. This model provides a clinical tool for real-time risk stratification, enabling personalized decision-making in post-endovascular therapy management, particularly regarding the timing of antithrombotic initiation and blood pressure control. However, further prospective validation in larger, multi-ethnic populations is necessary to confirm generalizability and improve model calibration.

## Data Availability

The raw data supporting the conclusions of this article will be made available by the authors, without undue reservation.

## Footnotes

1
https://github.com/PriorLabs/TabPFN/blob/main/examples/tabpfn_for_binary_classification.py
